# Analysis of the Prevalence of Binding and Neutralizing Antibodies against 39 Human Adenovirus Types in Student Cohorts Reveals Low-Prevalence Types and a Decline in Binding Antibody Levels during the SARS-CoV-2 Pandemic

**DOI:** 10.1128/jvi.01133-22

**Published:** 2022-11-07

**Authors:** Xiaoyan Wang, Leonie Kerkmann, Mario Hetzel, Sonja Windmann, Mirko Trilling, Wenli Zhang, Anja Ehrhardt, Wibke Bayer

**Affiliations:** a Institute for Virology, University Hospital Essen, University Duisburg-Essen, Essen, Germany; b Virology and Microbiology, Witten/Herdecke University, Witten, Germany; International Centre for Genetic Engineering and Biotechnology

**Keywords:** adenovirus, seroprevalence, neutralizing antibodies, binding antibodies, HAdV, adenoviruses, human adenovirus

## Abstract

Human adenoviruses (HAdVs) are important tools for vector development for applications such as immunization, oncolytic therapy, or gene therapy. However, their potential is limited by preexisting immunity against HAdV; therefore, it is important for future vector design to identify HAdV types of low seroprevalence. To provide such data, we performed an analysis of both binding and neutralizing antibodies in sera from three student cohorts. Among these young adults, we found the highest levels of binding antibodies against HAdV-C1, -D33, -A31, -B35, -C5, -D26, -E4, and -B7. The highest levels of neutralizing antibodies were detected against HAdV-C2, -B3, -C1, -F41, -G52, -C5, -A31, -E4, and -C6. While binding and neutralizing antibody levels were not different in males and females or in samples collected before and after the cold season, we found significantly lower levels of binding antibodies in sera collected 20 months after the beginning of the severe acute respiratory syndrome coronavirus 2 (SARS-CoV-2) pandemic, indicating a waning of HAdV-specific antibody responses on that time scale. Our data indicate that mainly HAdV types of species A, B, and D show low seroprevalence with regard to both binding and neutralizing antibodies and may represent good candidates for further characterization and future development as novel vector systems.

**IMPORTANCE** Vectors based on human adenoviruses (HAdVs) are important for the development of novel immunizations, oncolytic therapies, and gene therapies. The use of HAdV-based vaccines against Ebola virus, the rapid adaptation of the vector technology for vaccines against severe acute respiratory syndrome coronavirus 2 (SARS-CoV-2), and their very good efficacy have shown the great potential of HAdV-based vaccines. Preexisting immunity against HAdV-based vectors can limit their efficacy significantly; therefore, it is highly desirable to identify HAdV types with low seroprevalence. The identification of new suitable HAdV types for vector development will broaden the repertoire and contribute to future epidemic preparedness.

## INTRODUCTION

Human adenoviruses (HAdVs) were first described in 1953 when they were isolated from human adenoid tissue samples that showed cytopathic effects after prolonged *in vitro* cultivation by Rowe et al. (1) and were originally referred to as AD (adenoid-degenerating) or APC (adenoidal-pharyngeal-conjunctival) viruses, reflecting the finding that they were often isolated from adenoid tissue as well as from pharyngeal and conjunctival secretions of patients with respiratory infections or conjunctivitis (2).

Currently, 54 HAdV types have been recognized by the International Committee on Taxonomy of Viruses (ICTV) (3), which are grouped into 7 species, A through G, whereas 113 genotypes have been assigned by the research community in cooperation with the National Center for Biotechnology Information (4). It is important to note that HAdV types 1 through 54, which are recognized as distinct types by the ICTV, are immunologically sufficiently distinct to meet the ICTV type allocation criteria. These criteria include a low level of cross-neutralization with other types, with a ratio of homologous to heterologous titers of >16, or 8 to 16 if they exhibit sufficient further differences from other types in physical or biological properties. The pathogenesis of the HAdV depends on the type, and apart from the originally described respiratory and ocular infections, some HAdV types also cause infections of the gastrointestinal or urinary tract. Disseminated HAdV disease is rare in immunocompetent people but can be a serious complication in immunocompromised patients (reviewed in reference 5).

HAdVs are important tools for vector development for immunizations, gene therapy, and oncolytic therapy. Having been used for a long time in only experimental vector design, recent years brought important breakthroughs with the emergency use authorization of species D HAdV type 26 (HAdV-D26)-based vaccines against Ebola virus (6, 7) and, more recently, vaccines against severe acute respiratory syndrome coronavirus 2 (SARS-CoV-2) based on HAdV-C5 and HAdV-D26 (8, 9). It had been cautioned since the early days of HAdV-based vector development that the seroprevalence of HAdV-C5, the type on which vector systems were classically based, was relatively high and therefore likely to limit the widespread use of vectors based on this HAdV type (10). It was indeed demonstrated in clinical trials that a high level of preexisting antibodies would lead to impaired transgene-specific immune responses, especially CD8^+^ T cell responses (11, 12). Transgene-specific antibody responses have been shown to be less affected by antivector immunity (7, 13). Because of the impact of anti-HAdV immunity on the efficacy of HAdV-based vectors, clinical trials often include HAdV preimmunity-specific exclusion criteria. While many gene therapy or oncolytic vector trials exclude participants who were previously treated with HAdV-based vectors (14–19), others specified anti-HAdV neutralizing antibody (nAb) titers ranging from 1:320 to 1:1,000 as exclusion criteria (20–23). In HAdV-based vaccine studies, subjects have often been stratified according to their anti-HAdV neutralizing antibody titers, with neutralizing antibody titers of up to 1:45 (24) or 1:200 being regarded as low (7, 11, 12, 25). In one HIV vaccine study that combined DNA- and HAdV-C5-based immunization, only subjects with HAdV-C5-neutralizing antibody titers below 1:18 were included (26).

Many studies on the seroprevalence of HAdVs have been performed since their discovery. From an epidemiological point of view, seroprevalence provides important insight into how widespread the different HAdV types are. From the point of view of vector development, information on seroprevalence is crucial for the identification of suitable candidate types for the development of new HAdV-based vector systems that will not be impaired by preexisting anti-vector immunity. Initially, analyses were carried out mostly in acutely infected individuals, and increases in homotypic and heterotypic antibody responses were analyzed (27–29). In more recent years, many studies have been performed with healthy populations, with the aim of identifying HAdV types with low seroprevalence for HAdV-based vector development; a comprehensive review of these studies was reported by Mennechet et al. in 2019 (30). While most studies focused on a small number of HAdV types, there have also been two larger-scale studies that have analyzed the seroprevalence of a large number of HAdV types: Vogels et al. analyzed the prevalence of neutralizing antibodies against 51 HAdV types of species A through E in a cohort of Belgian adults (31), and D’Ambrosio et al. analyzed the prevalence of nAbs against 33 HAdV types, also of species A through E, in a cohort of Italian children and young adults (32). Taken together, these studies showed that the prevalence of neutralizing antibodies against the different HAdV types varied considerably, with the highest levels of reactivity being seen against species A, C, and E HAdV types, and there are significant regional differences.

In the present study, we evaluated the prevalence of both binding and neutralizing antibodies against 39 HAdV types in a cohort of young adults. To our knowledge, this is the first report of a side-by-side analysis of the prevalence of both binding and neutralizing antibodies against a large number of HAdV types covering species A through G.

## RESULTS

To analyze the seroprevalence of human adenovirus, we used 179 serum samples from a cohort of medical students. Serum samples were collected in October 2018 (*n* = 84) and April 2019 (*n* = 95). The cohorts comprised 57.1% (October 2018) and 64.2% (April 2019) females, and the ages of the serum donors ranged from 19 to 30 years (mean, 23.5 years) (October 2018) and from 21 to 37 years (mean, 24.3 years) (April 2019) (Table 1).

**TABLE 1:** Characteristics of the student cohorts

Characteristic	Value for group		
	October 2018 (*n* = 84)	April 2019 (*n* = 95)	October 2021 (*n* = 97)^*a*^
Mean age (yrs) (range)	23.5 (19, 30)	24.3 (21, 37)	23.0 (19, 40)
No. of donors of sex (%)			
Male	36 (42.9)	34 (35.8)	34 (35.1)
Female	48 (57.1)	61 (64.2)	63 (64.9)

a None of the serum donors had received an HAdV-based vaccine against SARS-CoV-2.

**FIG 1:**
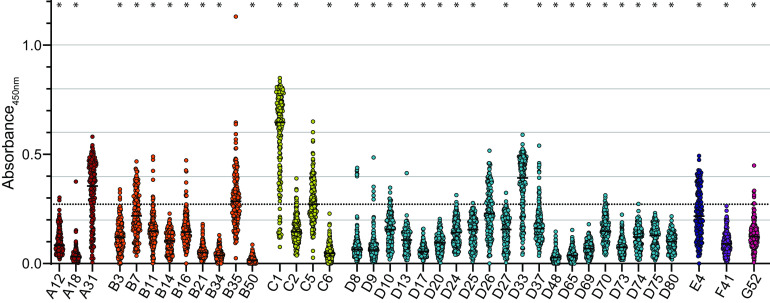
Binding antibody reactivity. Binding antibodies were analyzed using 179 serum samples collected in October 2018 and April 2019. Serum samples were diluted 1:1,000 in PBS and subjected to an HAdV binding antibody ELISA; absorbance values at a wavelength of 450 nm for each serum sample for the indicated HAdV types are shown. Each dot indicates an individual sample, and lines indicate the mean values for all serum samples for the indicated HAdV type. The dotted line indicates the mean value for HAdV-C5. * indicates a statistically significant difference compared to HAdV-C5 (*P < *0.05 by one-way ANOVA on ranks with Dunn’s post test). Data were generated in at least two independent experiments for each HAdV type; the data shown are from one representative experiment. All samples from October 2018 and April 2019 were analyzed side by side on the same plate for each HAdV type.

**FIG 2:**
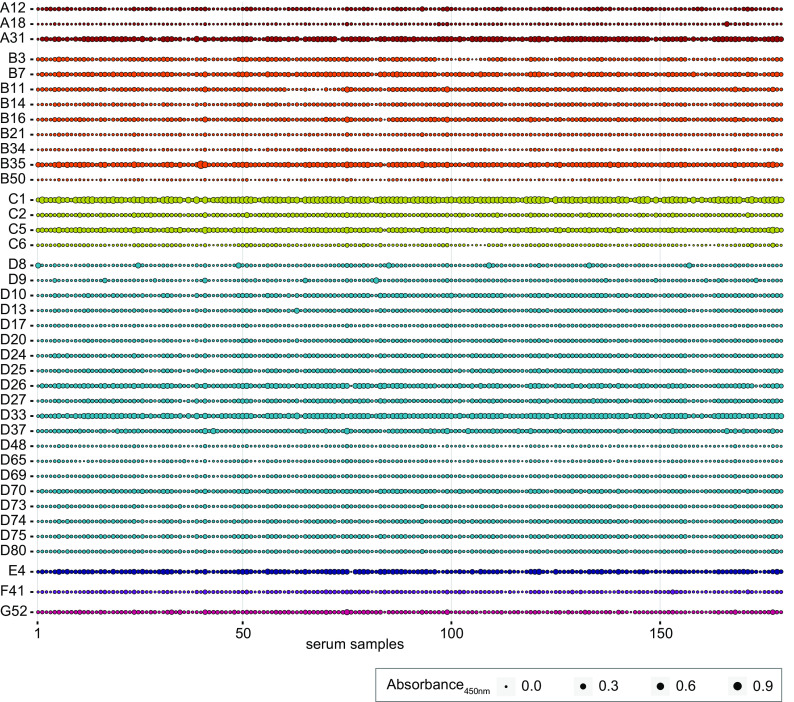
Reactivity of individual serum samples against the different HAdV types. The data shown in Fig. 1 for 179 serum samples collected in October 2018 and April 2019 are shown as a matrix bubble chart, showing reactivity against the indicated HAdV types for individual serum samples; the size of the dots indicates the absorbance value.

When we analyzed the levels of binding antibodies against adenoviruses, we found large variability in reactivity between the different adenovirus types, with the highest mean levels being observed for HAdV-C1, followed by HAdV-D33, -A31, -B35, -C5, -D26, -E4, and -B7 (in descending order), whereas the lowest reactivities were observed against HAdV-B50, -D48, -A18, -B34, -D65, -C6, -B21, and -D17 (in ascending order) (Fig. 1). Compared to the serum reactivity against HAdV-C5, which we regarded as the reference type since its use as a vector is regarded as problematic due to high preexisting immunity, we found significantly higher reactivity against HAdV-C1 and a tendency toward higher reactivity against HAdV-A31, -B35, and -D33, whereas the reactivity against all other tested HAdV types was lower. It is important to note that individual serum samples did not show universally low or high levels of binding antibodies across the different HAdV types, as illustrated in the matrix chart in Fig. 2.

**FIG 3:**
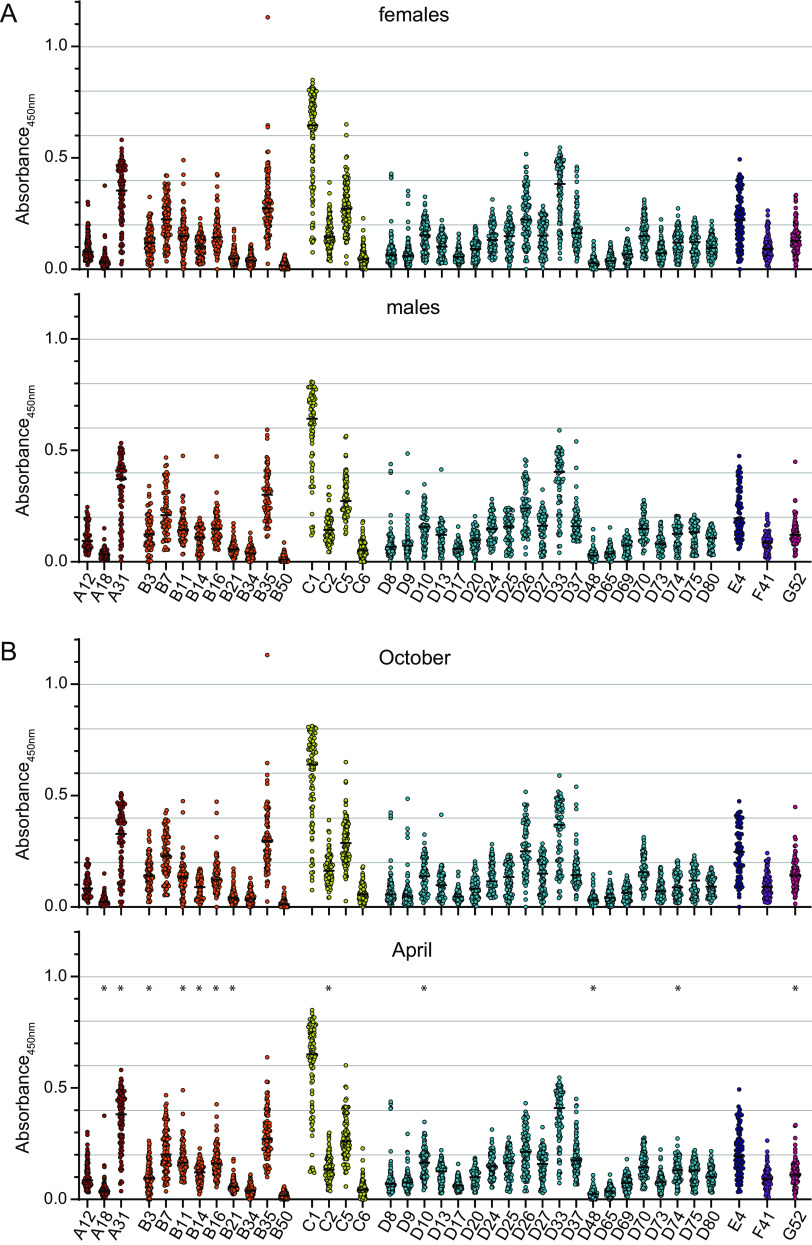
Stratification of binding antibody levels by sex of the donors and the time of serum collection. The data shown in Fig. 1 were stratified by sex, showing data from 70 male and 109 female serum donors (A), and by the time of sample collection, showing data from 84 serum samples from October 2018 and 95 serum samples from April 2019 (B). Each dot indicates an individual sample; lines indicate the mean values for the serum samples for the indicated HAdV type. * indicates statistically significant differences between values for serum samples collected in October and April (*P < *0.05 by an unpaired *t* test with Holm-Sidak multiple-comparison correction).

When we stratified the binding antibody data by the sex of the serum donors, we did not find any significant differences between males and females (Fig. 3A). Furthermore, we did not observe major changes in reactivity for samples collected in April 2019, i.e., after the cold season, compared to those collected in October 2018, although we did find small but significant increases in antibody levels against HAdV-A18, -A31, -B3, -B11, -B14, -B16, -B21, -D10, and -D74 and slight but significant decreases in antibody levels against HAdV-C2, -D48, and -G52 (Fig. 3B).

**FIG 4:**
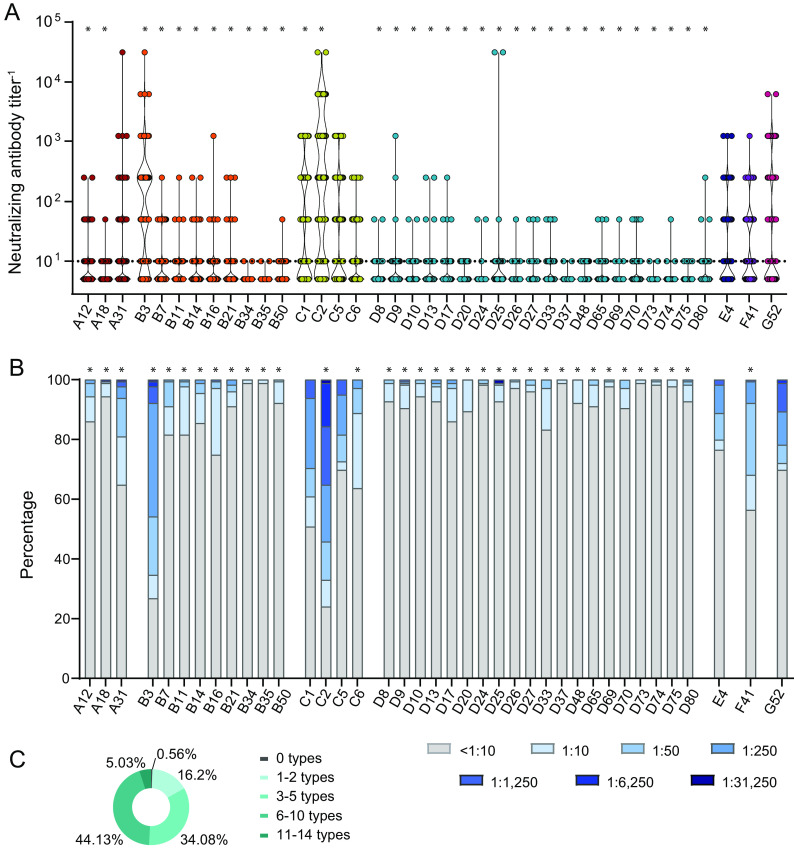
Neutralizing antibody levels and prevalence. Neutralizing antibody levels were analyzed using 179 serum samples from October 2018 and April 2019. (A) Serum samples were serially diluted, and the highest dilution that resulted in the neutralization of 90% of the input virus for each serum sample for the indicated viruses was determined. (B and C) Percentage of samples with the indicated levels of neutralizing antibodies against the indicated viruses (B) and percentage of serum samples that showed reactivity against the indicated number of HAdV types (C). The dotted line indicates the detection limit (A). * indicates a statistically significant difference compared to HAdV-C5 (*P < *0.05 by one-way ANOVA on ranks with Dunn’s post test [A] or a chi-squared test with Bonferroni multiple-comparison correction [B]). The data shown are from single analyses; duplicate assays were performed for confirmation.

We next analyzed the levels and prevalence of neutralizing antibodies against the same HAdV types in the sera of the student cohort, again using the serum samples from both October 2018 and April 2019. Interestingly, we found a quite different picture here since the virus types against which we found the highest reactivity were HAdV-C2, -B3, -C1, -F41, -G52, -C5, -A31, -E4, and -C6 (Fig. 4A and B). The majority of the individuals in the student cohort had only low or undetectable neutralizing antibodies against most of the other HAdV types, translating into significantly lower prevalences compared to HAdV-C5 (Fig. 4B). Interestingly, in some cases, we found very low overall neutralization levels but very high neutralizing activity for a few serum samples, such as for HAdV-D25.

Taken together, we found that 5% of the samples exhibited neutralizing activity against 11 or more of the tested HAdV types, and 44.1% showed neutralization of 6 to 10 HAdV types (Fig. 4C). Another one-third of the samples (34.1%) showed neutralizing activity against 3 to 5 types. There was only a single sample that did not show neutralizing activity against any of the tested HAdV types.

**FIG 5:**
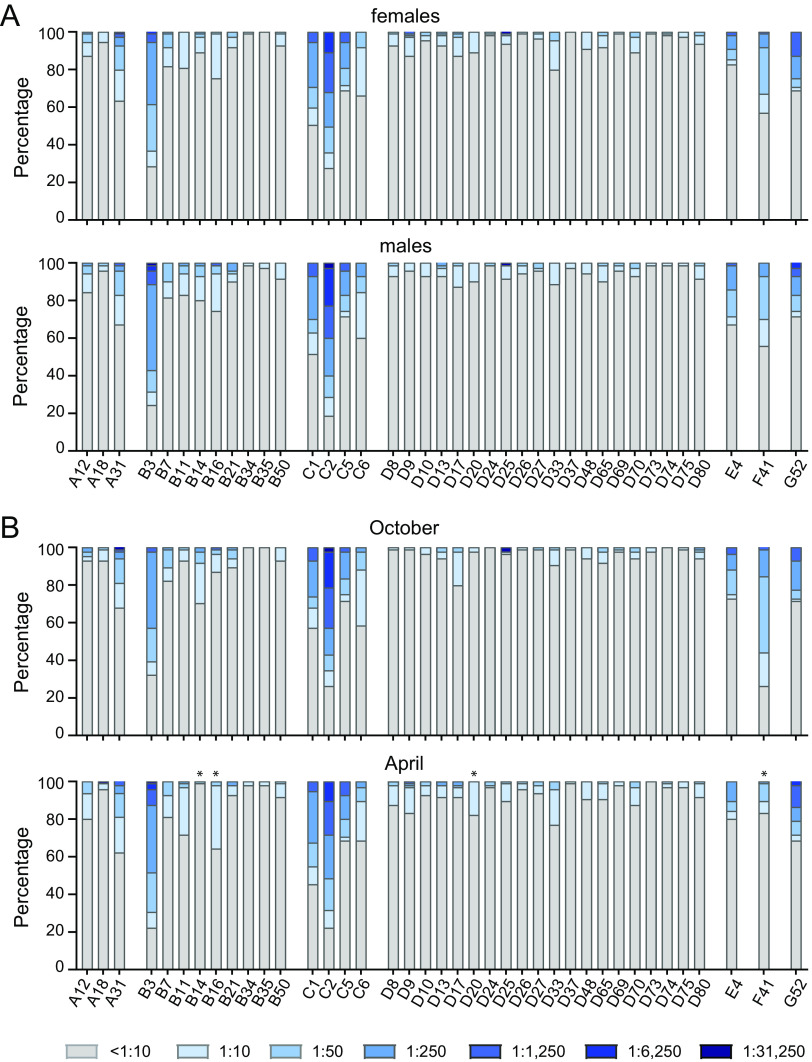
Stratification of neutralizing antibody prevalence by sex of the donors and the time of serum collection. The data shown in Fig. 4 were stratified by sex, showing data from 70 male and 109 female serum donors (A), and by the time of sample collection, showing data from 84 serum samples from October 2018 and 95 serum samples from April 2019 (B). * indicates statistically significant differences between values for serum samples collected in October and April (*P < *0.05 by a chi-squared test with Bonferroni multiple-comparison correction).

Also for the neutralizing antibodies, we did not detect significant differences between the neutralizing antibody levels observed in males and females (Fig. 5A). While the neutralizing antibody levels in samples collected in October 2018 and April 2019 were also largely comparable, a very noticeable difference was observed for the neutralization of HAdV-F41 (Fig. 5B), which was neutralized by <20% of the April serum samples, compared to >70% of the October serum samples.

Wondering if we would observe a decline in HAdV antibody levels in samples collected more recently, after almost 2 years of the SARS-CoV-2 pandemic that brought many restrictions to daily life, such as mask wearing and social distancing, which led to marked reductions in respiratory infections other than SARS-CoV-2 (33), we analyzed another 97 serum samples collected in October 2021. In this cohort, 64.9% of serum donors were females, and the mean age was 23.0 years, with a range of 19 to 40 years (Table 1). To rule out any influence of previous HAdV-based immunization on serum reactivity, we selected sample donors who had not received an HAdV-based vaccine against SARS-CoV-2.

**FIG 6:**
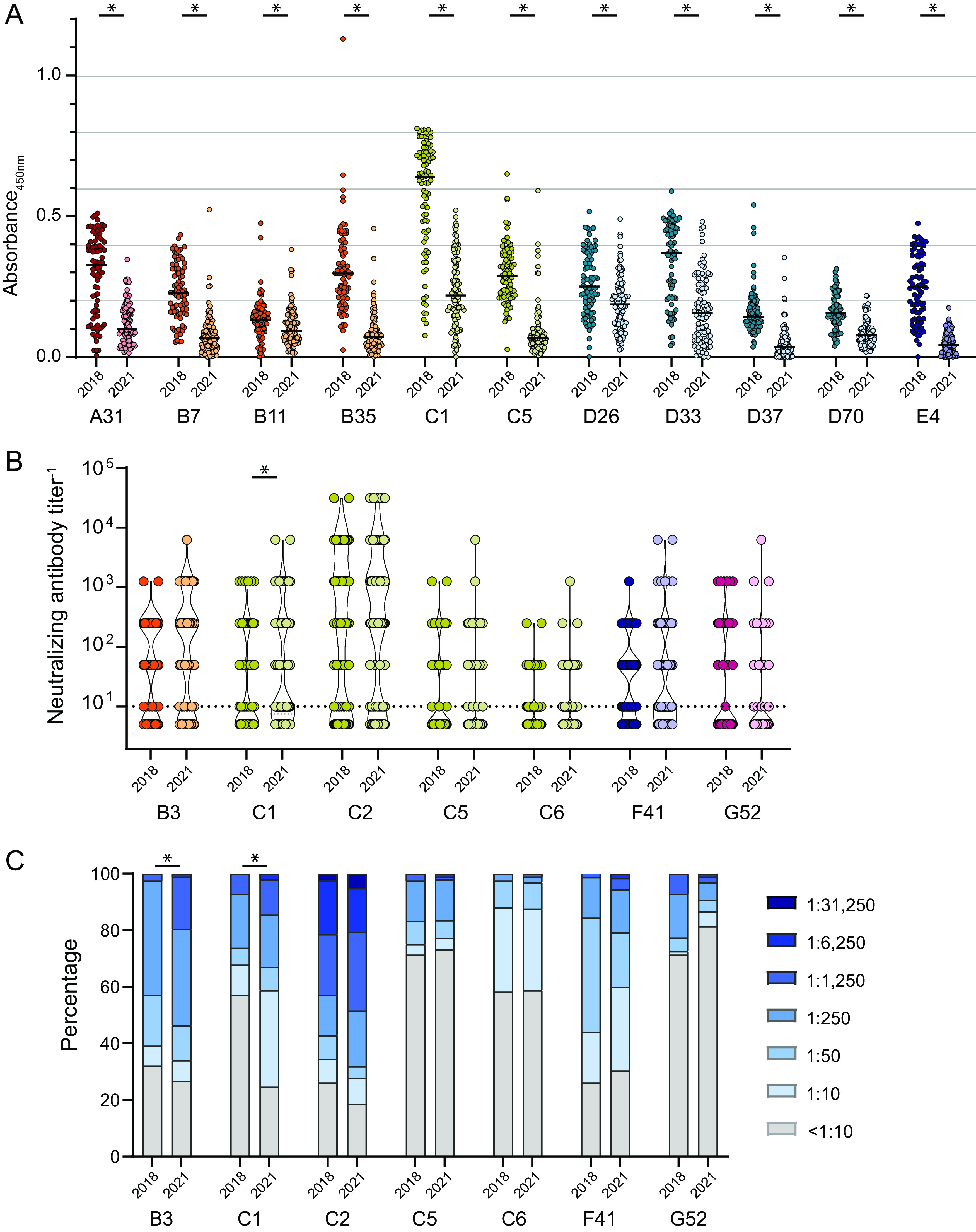
Comparison of reactivity before and during the SARS-CoV-2 pandemic. The binding (A) and neutralizing (B) antibody levels and the neutralizing antibody prevalences (C) of 84 serum samples collected in October 2018 and 97 serum samples collected in October 2021 were compared for the indicated viruses. For binding antibody analysis, all samples from October 2018 and October 2021 were analyzed side by side on the same plate for each HAdV type. Each dot indicates an individual sample (A and B), lines indicate the mean values for serum samples for the indicated HAdV types (A), and the dotted line indicates the detection limit (B). Statistically significant differences are indicated by * (*P < *0.05 by a Mann-Whitney rank sum test with Bonferroni multiple-comparison correction [A and B] or a chi-squared test with Bonferroni multiple-comparison correction [C]).

For the comparison of binding antibody levels, we chose the HAdV types for which we had observed the highest levels of reactivity with the previously obtained samples (Fig. 1). Interestingly, we found that the levels of binding antibodies for the October 2021 samples were consistently significantly lower than the binding antibody levels detected in samples from October 2018 (Fig. 6A). We also analyzed the levels of neutralizing antibodies in the serum samples from October 2021 against those for HAdV types that we had found to be most potently neutralized by the serum samples collected in 2018 and 2019. In contrast to the differences observed for the binding antibodies, the levels of neutralizing antibodies in the new serum samples were very similar to those found in the serum samples collected in 2018 and 2019. Interestingly, a slightly but significantly higher level of neutralizing antibodies was found for HAdV-C1 (Fig. 6B), and the prevalence of both HAdV-B3 and -C1 was found to be significantly higher in samples from October 2021 than in samples from October 2018 (Fig. 6C).

## DISCUSSION

Our data presented here give a comprehensive picture of the prevalence of both binding and neutralizing antibodies against a broad range of HAdV types of species A through G in healthy young adults. While we found high levels of reactivity against some HAdV types, we also found very little reactivity to many others, making them potential candidates for future HAdV-based vector development.

On the technical side, it has to be pointed out that not all HAdV types used in this study had been purified by the same method. While most preparations were purified by a CaptoCore bead-based method, a few preparations were purified by cesium chloride gradient ultracentrifugation or by using the column-based AdenoPack kit (Table 2). The presence of larger amounts of empty particles in CaptoCore- or AdenoPack-purified preparations than with cesium chloride gradient purification might result in stronger signals in the enzyme-linked immunosorbent assay (ELISA) and reduced apparent titers in the neutralization assay. However, a side-by-side comparison of an HAdV-C5-based vector purified by any of the three methods revealed no differences in absorbance values for the tested serum samples when the three different preparations were used for ELISA plate coating (data not shown), indicating that there is no major influence of the different purification methods on the outcome of our study. Similarly, there is some degree of variation in the ratio of the viral particle concentration to the tissue culture infectious dose (Table 2). While the presence of larger numbers of noninfectious particles might lead to a reduced signal by intracellular ELISA (icELISA) staining, the internal controls of 10^6^ and 10^5^ viral particles (vp) that are carried on each assay plate serve as the internal reference, ensuring the reliability of the assay.

**TABLE 2:** Characteristics of the HAdV types and conditions of the neutralization icELISA

Species	Type	Purification method	Concentration		Cell line	Incubation time (h)
			vp/mL (OD_260_)	TCID_50_/mL		
A	12	CaptoCore	1.54 × 10^12^	4.21 × 10^9^	293A	66
	18	CaptoCore	1.59 × 10^12^	1.78 × 10^8^	293A	66
	31	CsCl	2.70 × 10^12^	3.16 × 10^7^	293A	90

B	3	CaptoCore	1.84 × 10^12^	4.40 × 10^10^	A549	48
	7	CaptoCore	6.35 × 10^12^	1.78 × 10^10^	A549	40
	11	CaptoCore	3.61 × 10^12^	1.0 × 10^12^	A549	66
	14	CaptoCore	2.44 × 10^13^	3.16 × 10^12^	A549	48
	16	CaptoCore	5.82 × 10^12^	1.33 × 10^8^	293A	66
	21	CaptoCore	8.91 × 10^11^	4.39 × 10^9^	A549	66
	34	CaptoCore	6.64 × 10^12^	3.16 × 10^12^	A549	48
	35	CaptoCore	1.46 × 10^12^	1.33 × 10^12^	A549	40
	50	AdenoPack	1.23 × 10^13^	4.22 × 10^9^	293A	66

C	1	CaptoCore	4.24 × 10^12^	3.16 × 10^12^	A549	26
	2	CaptoCore	1.14 × 10^13^	4.39 × 10^8^	A549	40
	5	CaptoCore	2.13 × 10^12^	2.28 × 10^11^	293A	40
	6	CaptoCore	1.96 × 10^12^	1.33 × 10^14^	293A	66

D	8	CsCl	1.2 × 10^11^	7.5 × 10^7^	293A	114
	9	CaptoCore	9.67 × 10^12^	6.17 × 10^9^	A549	66
	10	CaptoCore	3.03 × 10^13^	3.16 × 10^12^	A549	66
	13	CaptoCore	2.42 × 10^12^	1.0 × 10^10^	A549	66
	17	CaptoCore	3.8 × 10^12^	5.6 × 10^11^	293A	66
	20	CaptoCore	2.99 × 10^13^	3.16 × 10^12^	A549	66
	24	CaptoCore	1.29 × 10^12^	1.17 × 10^10^	A549	66
	25	CaptoCore	7.37 × 10^12^	3.16 × 10^11^	A549	40
	26	CaptoCore	2.87 × 10^12^	3.16 × 10^12^	A549	40
	27	CaptoCore	6.5 × 10^12^	3.16 × 10^12^	A549	40
	33	CaptoCore	1.62 × 10^12^	3.16 × 10^10^	A549	138
	37	CaptoCore	3.12 × 10^12^	4.39 × 10^11^	A549	66
	48	AdenoPack	7.7 × 10^12^	2.37 × 10^10^	293A	66
	65	CaptoCore	1.15 × 10^13^	4.36 × 10^11^	293A	66
	69	CaptoCore	5.45 × 10^12^	1.17 × 10^12^	293A	40
	70	CaptoCore	9.85 × 10^12^	1.17 × 10^12^	A549	40
	73	CaptoCore	1.91 × 10^12^	1.64 × 10^12^	A549	40
	74	CaptoCore	1.97 × 10^12^	6.15 × 10^10^	A549	40
	75	CaptoCore	2.85 × 10^12^	8.51 × 10^11^	A549	40
	80	CaptoCore	2.83 × 10^12^	8.48 × 10^11^	A549	40

E	4	CaptoCore	2.17 × 10^12^	4.37 × 10^9^	A549	66

F	41	CsCl	4.2 × 10^11^	7.5 × 10^8^	293A	90

G	52	CaptoCore	4.36 × 10^12^	3.16 × 10^12^	293A	66

It has to be noted that we did not always observe a good correlation between binding and neutralizing antibodies against the individual HAdV types. For some types such as HAdV-B35, -C1, and -D33, neutralizing antibody levels were lower than suggested by the binding antibody levels, whereas the high degree of neutralization of HAdV-C2 was far more pronounced than expected from the relatively mediocre binding antibody levels. These findings suggest that either many types induce mainly nonneutralizing antibodies, resulting in the discrepancy of relative binding and neutralizing antibody levels, or there is a high contribution of cross-reactivity for the binding antibodies, or both.

Since there are relatively few data on the prevalence of binding antibodies in healthy individuals, it is difficult to compare our data with those from other studies. Most studies on the seroprevalence of HAdV have focused on the presence of neutralizing antibodies. It should be highlighted that previous results from the mouse model suggest that an impairment of transgene-specific immune responses can already be observed with low levels of vector-neutralizing antibodies, suggesting that nonneutralizing, binding antibodies against the HAdV vector may also play a role in the vaccination outcome (34). It is interesting to note that the prevalence of neutralizing antibodies against many types, including HAdV-C5, was not as high in our cohort as had been reported previously. While comparison is sometimes hindered by the fact that studies worked with different lowest dilutions, it is clear that the ranking of the HAdV types with regard to their seroprevalence gave a different picture in many other studies. In the large study by Vogels et al. (31), the highest frequencies of serum samples were reactive against, in descending order, HAdV-C5, HAdV-C1, HAdV-A31, HAdV-A12, HAdV-B3, and HAdV-C2; almost 80% of all tested serum samples were reactive against HAdV-C5. This is in contrast to our data: while we also saw a high frequency of serum samples with neutralizing activity against HAdV-B3, we saw even more reactivity against HAdV-C2 but far less reactivity against HAdV-C5, against which only 30% of the serum samples of our student cohort showed neutralizing activity.

Our cohort consisted of only young adults, and we cannot extrapolate an estimate of seroprevalence to other cohorts such as children or the elderly. It may be speculated that the number of HAdVs that are seroprevalent increases with age due to the accumulation of infections over time. It was found in an early report by Huebner et al. in 1954 from a comparison of serum samples from children and young adults that different age groups may have very different prevalences of antibodies to specific types. Those authors found that only 25% of children between 6 and 15 years of age had neutralizing antibodies against HAdV-E4, compared to 70% of young adults aged 16 to 34 years, which suggested that different HAdVs are not always in wide circulation (27). The study by D’Ambrosio et al. (32) showed in a comparison of children of different age groups and young adults that for most viruses, the frequency of neutralizing sera increased in young children during the first years of life, although those authors also found rather high levels of probably maternal antibodies in very young children less than 6 months of age. An increase in the frequency of neutralizing sera in adults compared to older children was observed for only some of the analyzed viruses, such as HAdV-C2, -C5, -C6, and -B21, while the frequency was similar or actually reduced for other HAdV types such as HAdV-C1, HAdV-B3, HAdV-E4, and HAdV-A12 (32). This finding confirms the theory by Huebner et al. that some HAdV types are in continuous circulation, leading to a steady increase in seroprevalence with age, whereas other types are not always in circulation, and seropositivity may actually be lower in older individuals.

For similar reasons, it seems to only partially hold true that seroprevalence against HAdV increases over the winter months. Our data generally show only minor differences, although this has to be interpreted with caution since we did not collect serial samples from the same individuals at different time points. One study analyzed the prevalence of binding antibodies against HAdV-1 through HAdV-6 as mixed antigens in a general population in the winter and spring of 1954 to 1955 and demonstrated the highest levels in samples collected in February compared to the other months (35); also, in this case, samples were obtained from different individuals at different time points.

On the other hand, our data indicate that a longer period of social distancing and mask wearing, which led to a marked reduction in common cold cases (33), also resulted in a decline in binding antibody levels against the 11 HAdV types tested for this comparison. It must not be overlooked, however, that this was not true for the levels of neutralizing antibodies, and overall, this finding has to be taken with some caution since we analyzed samples from different donors. For a more detailed analysis of the dynamics of antibody levels with time, longitudinal sample collection over a longer time from a specified cohort would be necessary.

It has to be noted that our seroprevalence data are of course not representative of global seroprevalence. It has been shown multiple times previously that seroprevalence can be much higher in different countries, for example, in Africa and Asia compared to Europe and the United States (36–40), and may also differ within countries, as has been shown for the seroprevalence of HAdV-C5 in different regions of China (41). Therefore, it would be important to also analyze sera from other regions of the world against this large panel of HAdV types to provide a more universal picture.

It is also important to keep in mind that the prevalence of binding and neutralizing antibodies in sera does not allow a direct conclusion on the spread of individual viruses since there is only limited information on the cross-reactivity of HAdV-induced antibodies. Some information can be drawn from studies of antibody responses after HAdV infection where antibodies were determined at the time of acute infection and after recovery. Grayston et al. analyzed the neutralization of HAdV-C1 through HAdV-D10 and showed that sera from HAdV-B3-infected individuals showed an increase in reactivity against HAdV-C2; sera from HAdV-E4-infected individuals showed an increase in reactivity against HAdV-B3, -B7, -C5, and -C6; and sera from HAdV-B7-infected individuals showed an increase in reactivity against HAdV-B3, -E4, -C5, -C6, -D8, -D9, and -D10 (28). Furthermore, those researchers used sera from experimentally infected rabbits and demonstrated some neutralizing reactivity of HAdV-C2-induced sera against HAdV-B3, HAdV-C6-induced sera against HAdV-C5, and HAdV-B3-induced sera against HAdV-B7 and vice versa, of HAdV-D9-induced sera against both HAdV-D8 and HAdV-E4 and of HAdV-D10-induced sera against HAdV-D9. A thorough understanding of the cross-reactivity of binding and neutralizing antibodies requires a detailed analysis of many HAdV-type-specific serum samples against a broad range of HAdV types, which will be performed in the future.

In conclusion, our data show that especially viruses from species A, B, and D show very low seroprevalence, which makes them interesting candidates for the development of either novel HAdV-type-based vectors or capsid-chimeric vectors in combination with previously used HAdV types. Besides seroprevalence, an important factor to consider for the selection of individual HAdV types for future vector development is the immunogenicity of the viruses, which will also influence the strength of transgene-specific immune responses, and this will be addressed in detail in future studies. Based on the presented data, we suggest that those HAdV types for which we found both low neutralizing antibody prevalence and low binding antibody levels should be considered the most favorable candidates for vector development, such as HAdV-A12, -A18, -B21, -B34, -B50, -D8, -D9, -D17, -D48, -D65, -D69, and -D73.

## MATERIALS AND METHODS

### Cells.

293A (Microbix Biosystems, Toronto, ON, Canada) and A549 (ATCC CCL-185; LGC Standards, Wesel, Germany) cells were propagated in Dulbecco’s modified Eagle medium (DMEM) with high glucose supplemented with 10% heat-inactivated fetal bovine serum, 50 μg/mL gentamicin (all cell culture reagents were obtained from Invitrogen-Gibco, Thermo Fisher Scientific, Karlsruhe, Germany), and 20 μg/mL ciprofloxacin (Fresenius Kabi, Austria). Cells were maintained in a humidified 5% CO_2_ atmosphere at 37°C.

### Viruses and viral vectors.

The HAdV-C5 (ATCC VR-5) and HAdV-F41 (ATCC VR-930) strains were obtained from the American Type Culture Collection (LGC Standards, Wesel, Germany). HAdV-A12, -A18, -A31, -B3, -B21, -B11, -B14, -B35, -C6, -D9, -D10, -D13, -D17, -D20, -D24, -D25, -D26, -D27, -D33, -D37, and -E4 were clinical isolates provided by Gundula Jäger and Hans Nitschko (Department of Virology, Max von Pettenkofer Institute, Ludwig Maximilian University, Munich, Germany). HAdV-B7, -B34, -C1, -C2, -D8, and -G52 were kindly provided by Thomas Dobner (Heinrich Pette Institute, Leibnitz Institute for Experimental Virology, Hamburg, Germany). HAdV-D70, -D73, -D74, -D75, and -D80 were kindly provided by Albert Heim (Institute for Virology, Hannover Medical University, Hannover, Germany). The identity of all HAdV types was verified by sequencing.

The vectors Ad16.GLN, Ad65.GLN, and Ad69.GLN encode enhanced green fluorescent protein (eGFP) and luciferase and were constructed as described previously (42). The eGFP-encoding vector Ad48.GFP and the luciferase-encoding vector Ad50.Luc were constructed using the pAdApt system as described previously (43).

The viruses were propagated on 293A or A549 cells (Table 2). Viruses were purified using the standard cesium chloride gradient-based ultracentrifugation method (42) or the Vivapure AdenoPack 20 kit (Vivascience, Hannover, Germany) or by an in-slurry CaptoCore purification method adapted from methods described previously (44). For CaptoCore purification, AdV-infected cells were harvested when cytopathic effect had developed and all cells were rounded but not yet lysed. Cells were harvested and pelleted for 10 min at 1,000 × *g*, and the cell pellet was resuspended in 10 mM Tris buffer (pH 8.0). After three cycles of freezing and thawing, cells were centrifuged as described above, and the supernatant was transferred to a new tube. Four hundred microliters of the CaptoCore slurry (Sigma-Aldrich, Taufkirchen, Germany) was washed two times with 10 mM Tris buffer, resuspended in 200 mL of 10 mM Tris buffer, and transferred to the AdV lysate. The samples were mixed end over end for 45 min at room temperature and centrifuged for 10 min at 800 × *g*, and the supernatant was transferred to a new tube. The incubation step with fresh CaptoCore was repeated two times; the final supernatant was diluted in a solution containing 20 mM Tris-HCl, 25 mM NaCl, and 2.5% glycerol (pH 8.0) and concentrated over VivaSpin columns (Sartorius Stedim Biotech, Göttingen, Germany).

The AdV particle concentrations (viral particles [vp] per milliliter) were determined by spectrophotometry as described previously (45). The infectivity of the virus preparations was verified by a 50% tissue culture infectious dose (TCID_50_) assay.

### Serum samples and ethics approval.

Serum samples were collected from cohorts of students; the characteristics of these student cohorts are shown in Table 1. Serum samples and serum dilutions were stored at −20°C. The students provided information on their age and, in October 2021, on any SARS-CoV-2 vaccines received and voluntarily gave their informed consent to the storage of their serum samples and the use thereof after anonymization for the study of HAdV-specific antibodies for research purposes and to the publication of the obtained results. This study was approved by the Ethics Committee of the Medical Faculty of the University of Duisburg-Essen (18-8147-BO).

### Binding antibody ELISA.

The binding antibody ELISA was performed in 384-well Nunc MaxiSorp plates (Sigma-Aldrich, Taufkirchen, Germany). Plates were coated with 2.5 × 10^7^ vp of purified, UV-inactivated AdV in 25 μL phosphate-buffered saline (PBS) per well overnight at 4°C. Plates were washed with PBS plus 0.1% Tween 20 (PBS-T) and blocked with 75 μL/well PBS plus 20% fetal calf serum (FCS) for 5 h at room temperature. Twenty microliters of serum samples diluted 1:1,000 in PBS was added to the wells after discarding the blocking buffer, and the plates were incubated at 4°C overnight. Binding antibodies were detected using 25 μL/well of a horseradish peroxidase-labeled polyclonal donkey anti-human IgG antibody (Dianova, Hamburg, Germany) diluted 1:15,000 in PBS and 25 μL of tetramethylbenzidine (TMB) as the substrate (1-step TMB Ultra; Thermo Fisher, Waltham, MA, USA) per well, with washing using PBS-T between steps. The substrate reaction was stopped by the addition of 25 μL 1 N H_2_SO_4_, and the absorption at 450 nm was analyzed using a Mithras^2^ microplate reader (Berthold Technologies, Bad Wildbad, Germany).

### Neutralizing antibody assay.

To detect HAdV-neutralizing antibodies, serial dilutions of student serum samples from 1:10 to 1:31,250 in 5-fold dilution steps were prepared with DMEM. Twenty-five microliters of the respective dilutions were mixed with different HAdV types at a concentration of 10^6^ vp/25 μL in 96-well flat-bottom plates. Control reactions were set up with adenovirus concentrations of 10^6^ vp/25 μL and 10^5^ vp/25 μL, which were mixed with 25 μL of medium without human serum and carried along as five replicates each on each plate. The reaction mixtures were incubated at 37°C for 60 min, and 1 × 10^4^ 293A or A549 cells were added to the plates and incubated for 26 to 138 h (Table 2). Different incubation times were necessary to accommodate the different speeds of replication of the individual HAdV types and the different efficacies of detection by the detection antibodies; optimal incubation conditions that allowed the easy distinction of infection by 10^6^ and 10^5^ vp were chosen for each HAdV type. For the detection of HAdV in the assay plates, cells were fixed with a 4% paraformaldehyde (PFA) solution for 30 min at room temperature, followed by 60 min at 37°C (293A cells), or with 2% PFA for 30 min at room temperature, followed by 30 min at 37°C (A549 cells). Cells were permeabilized with 1% Ecosurf (Thermo Fisher Scientific, Waltham, MA) in PBS for 30 min and blocked with 10% FCS in PBS for 1 h at room temperature. Afterward, 50 μL of anti-adenovirus hexon antibody 8C4 (BioConnect, Huissen, Netherlands) diluted 1:5,000 in blocking buffer was added to each well, and the mixture was incubated overnight at 4°C. After washing the plates 3 times with PBS-T, 50 μL of a horseradish peroxidase-labeled rabbit anti-mouse antibody (Dako Agilent, Santa Clara, CA) diluted 1:10,000 in blocking buffer was added to each well, and the mixture was incubated for 2 h at room temperature, followed by 5 washes with PBS-T. Subsequently, 50 μL of the TMB Ultra substrate was added to each well. The reaction was stopped by the addition of 50 μL 1 N H_2_SO_4_, and the optical density at 450 nm (OD_450_) was read using the Mithras^2^ microplate reader. Plasma dilutions were considered neutralizing when more than 90% of the input virus was neutralized, i.e., when the values obtained for the dilution were lower than those for the 10^5^-vp control.

### Statistical analysis.

Statistical analyses were performed in GraphPad Prism 8.4.2 using the unpaired *t* test with Holm-Sidak correction for multiple comparisons, the Mann-Whitney rank sum test with Bonferroni correction for multiple comparisons for pairwise comparisons of antibody levels, or one-way analysis of variance (ANOVA) on ranks for comparisons of antibody levels among multiple groups. A chi-squared test with Bonferroni correction for multiple comparisons was performed for comparisons of antibody prevalences.
